# Multicentric Castleman disease (MCD) presenting with retiform purpura: A rare dermatologic manifestation of a systemic disease

**DOI:** 10.1016/j.jdcr.2025.12.036

**Published:** 2025-12-29

**Authors:** Shamey Kassim, Aisha Jamison, Lindsey Warner, Michi M. Shinohara, Mariam Alam, Anna L. Cogen

**Affiliations:** a University of Washington School of Medicine, Seattle, Washington; b Department of Dermatology, University of Washington, Seattle, Washington

**Keywords:** Castleman Disease, Cutaneous vasculopathy, Retiform Purpura, TAFRO syndrome

## Introduction

Multicentric Castleman disease (MCD) is a heterogenous group of systemic lymphoproliferative disorders characterized by generalized lymphadenopathy and multiorgan involvement.^1^ While MCD can be associated with human herpesvirus-8 (HHV-8) and immunocompromise, many cases are idiopathic.^2^ MCD is described as a “great imitator” given its varied presentations that may mimic systemic autoimmune diseases and benign and malignant adenopathies.^2^ The diagnostic complexity of MCD frequently contributes to delays in recognition.^2^

Retiform purpura is characterized by net-like non-blanching purple patches arising from microvascular injury or occlusion.^3^ While retiform purpura elicits a broad differential, its association with MCD has not been previously reported. MCD has, however, been associated with non-retiform purpura in the setting of thrombotic thrombocytopenic purpura, immune-mediated thrombocytopenia and IL-6 mediated thrombosis, pointing toward possible cutaneous sequelae of coagulopathy in MCD and associated disorders. We present a case highlighting an unusual presentation of retiform purpura with subsequent development of purpura fulminans in the setting of MCD and acute thrombotic microangiopathy (TMA).

## Case presentation

**Fig 1:**
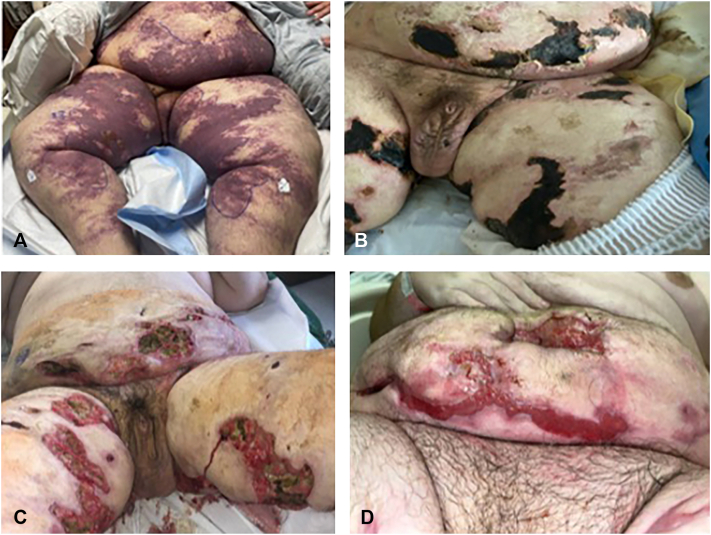
Progression of purpuric and necrotic skin lesions. Multiple non-blanching, purpuric patches with overlying bullae (**A**) on the inner thighs, abdomen, and back, which progressed to stellate necrotic plaques (**B**), with subsequent ulceration (**C**) and then wound healing with granulation tissue (**D**) in the setting of wound care, IV sodium thiosulfate, silutximab, and operative debridement.

A 26-year-old male with history of hypertension, obesity, and acute renal failure of unclear etiology requiring hemodialysis 6 months prior to presentation presented with abdominal pain, nausea, vomiting, and shortness of breath. Initial laboratory evaluation showed acute kidney injury (creatinine 5.9 mg/dL), microscopic hematuria, moderate proteinuria, microcytic anemia (hemoglobin 9.4 g/dL) and thrombocytopenia (platelets 75 K/μL). Further workup revealed elevated haptoglobin (449 mg/dL), normal lactate dehydrogenase, low ADAMTS13 activity (55%, normal >67%), negative antinuclear antibody, elevated IL-6 (149, normal 0-6 pg/mL), elevated methylmalonic acid (0.56 mcmol/L), an absence of schistocytes on peripheral smear, and negative stool Shiga toxin. Renal biopsy confirmed TMA. The patient was treated with meningococcal vaccination, eculizumab (complement C5 inhibitor) and penicillin prophylaxis. Despite these treatments, he developed renal failure requiring hemodialysis, shock, and violaceous reticulated patches with overlying bullae on the abdomen and bilateral thighs (Fig 1). Radiographic imaging demonstrated widespread adenopathy and hepatosplenomegaly. Core needle lymph node biopsy showed reactive changes and no evidence of malignancy.

**Fig 2:**
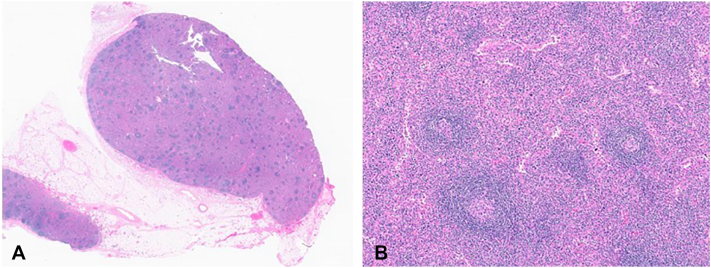
Excisional lymphnode biopsy. H&E stained slides demonstrating atretic lymphoid follicles (**A,** 0.5×) and a higher power view of atretic lymphoid follicles with thickened mantle zones, and interfollicular vascular proliferation (**B**, 5×).

**Fig 3:**
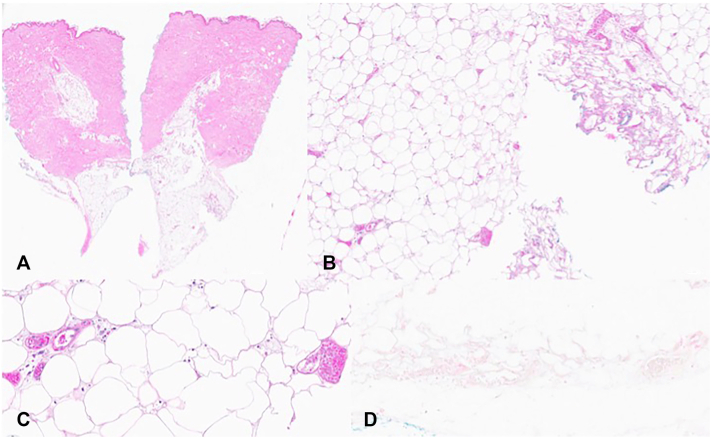
Lesional biopsy showing deep vascular fibrin deposition. H&E stained slides demonstrating a punch biopsy specimen to the depth of the subcutis (**A**, 10×). Higher power sections showing fibrin deposition in association with deeper small vessels of the dermis and subcutis (**B**, 40×, and **C**, 150×). The Van Kossa stain was negative for calcium deposition (**D**, 100×).

Excisional lymph node biopsy from the left axilla confirmed HHV-8 negative MCD (Fig 2). The patient met criteria for idiopathic multicentric Castleman disease (IMCD), specifically the thrombocytopenia anasarca fever reticulin fibrosis/renal dysfunction and organomegaly syndrome (TAFRO) subtype, characterized by thrombocytopenia, anasarca, fever, renal dysfunction, and organomegaly. He received methylprednisolone 500 mg daily for 5 days, followed by an oral prednisone taper, and 3 doses of siltuximab. The purpura progressed rapidly, with further blistering and secondary ulcerations during the course of his 4-week admission. Scintigraphy showed increased radiotracer uptake in the subcutaneous soft tissues, suggestive of calcium deposition. Skin biopsy revealed deep vascular fibrin deposition concerning for a thromboembolic process (Fig 3). Laboratory results showed normal fibrinogen (217 mg/dL), prothrombin time (15.3 sec), international normalized ratio (1.2), partial prothrombin time (28 sec), with elevated D-dimer (16.77), haptoglobin (358 mg/dL), and a weakly positive lupus anticoagulant (6.5). Antiphospholipid antibodies (anti-cardiolipin and anti-β2 glycoprotein I) were negative. The patient received temporary sodium thiosulfate (STS) for suspected calciphylaxis, surgical debridement, a 7-day course of cephalexin and levofloxacin for a secondary skin soft tissue infection and enoxaparin.

The patient’s wounds continued to worsen, and STS was discontinued. Sepsis was considered given the patient’s initial shock presentation and skin findings. He received empiric broad-spectrum antibiotics, but blood cultures remained negative and no infectious source was found. His lack of response to antimicrobials and subsequent improvement with MCD-directed therapy supported a non-infectious etiology. Although he met laboratory criteria for disseminated intravascular coagulation, coagulopathy was attributed to cytokine-mediated endothelial injury from MCD.

Given the rapid progression of his cutaneous findings with evidence of skin necrosis and concomitant coagulopathy, purpura were attributed to purpura fulminans. He remained on a prolonged steroid taper for treatment of MCD, received diligent wound care, anticoagulant therapy, and periodic surgical debridements. His ulcers began to improve over the course of 6 weeks. The patient was ultimately transferred to a long-term acute care facility for complex wound care.

## Discussion

While retiform purpura has not been previously associated with MCD, the disease pathophysiology provides a plausible mechanism. MCD involves dysregulated cytokine production with dramatically elevated IL-6, vascular endothelial growth factor, and fibrinogen leading to systemic inflammatory symptoms and lymphadenopathy.^4^ Overproduction of IL-6 directly affects vascular endothelial cells, leading to production of proinflammatory cytokines and chemokines, increased vascular permeability, and activation of the coagulation cascade.^5^ These effects contribute to endothelial dysfunction, leading to microvascular injury and thrombosis in MCD.^5^ Patients with IMCD-TAFRO may exhibit transfusion-resistant thrombocytopenia and large platelets, resembling immune-mediated platelet destruction seen in immune thrombocytopenic purpura, suggesting an autoimmune mechanism underlying thrombocytopenia in some patients with IMCD-TAFRO.^6^

We suspect the systemic inflammatory environment of MCD contributed to the development of retiform purpura in this patient, in concert with his known TMA. TMA refers to a group of conditions characterized by microvascular changes, including thrombosis, in association with microangiopathic hemolytic anemia, thrombocytopenia, and organ injury.^7^ Cutaneous manifestations of TMA are driven by vascular occlusion and subsequent ischemia. Retiform purpura may occur in the setting of TMA due to the shared underlying vascular pathology, and certain forms of TMA have been described in literature presenting with purpura.^3^^,^^8^ In this patient, the observed retiform purpura was likely at least in part due to TMA with cutaneous microthrombi formation, and it is possible that TMA was the intermediary step between systemic inflammation in MCD and development of retiform purpura.

This case highlights a rare co-occurrence of IMCD (IMCD-TAFRO) and retiform purpura progressing to purpura fulminans in the setting of TMA. While a causal relationship cannot be established from a single case with overlapping pro-thrombotic conditions, the findings raise the possibility that cytokine-mediated endothelial injury in MCD may contribute to retiform purpura development. This case is also notable for successful healing of retiform purpura, which has not been extensively described in the literature, and represents a promising course for patients with retiform purpura.

## Conclusion

This case highlights the rare co-presentation of MCD and retiform purpura. Although retiform purpura is a well-known clinical finding with a broad differential, an association with MCD has not been widely documented. This case emphasizes the importance of including MCD in the differential diagnosis of retiform purpura in cases associated with lymphadenopathy, elevated inflammatory markers, and end-organ dysfunction. This case also contributes to the limited literature on the natural history of retiform purpura by describing a case of cutaneous recovery.

## Conflicts of interest

None disclosed.
